# Enhancing community preparedness: an inventory and analysis of disaster citizen science activities

**DOI:** 10.1186/s12889-019-7689-x

**Published:** 2019-10-23

**Authors:** Ramya Chari, Elizabeth L. Petrun Sayers, Sohaela Amiri, Mary Leinhos, Virginia Kotzias, Jaime Madrigano, Erin V. Thomas, Eric G. Carbone, Lori Uscher-Pines

**Affiliations:** 1 RAND Corporation, 1200 South Hayes Street, Arlington, VA 22202 USA; 2 RAND Corporation, 1776 Main Street, Santa Monica, CA 90401 USA; 3 Center for Preparedness and Response, Centers for Disease Control and Prevention, 2877 Brandywine Road, MS K-72, Atlanta, GA 30341 USA; 4 Center for Preparedness and Response, Centers for Disease Control and Prevention, 1600 Clifton Road NE, MS K-72, Atlanta, GA 30333 USA

**Keywords:** Citizen science;public health emergency preparedness, Disaster, Inventory, Disaster resilience, Emergency response, Disaster recovery

## Abstract

**Background:**

Disaster citizen science, or the use of scientific principles and methods by “non-professional” scientists or volunteers, may be a promising way to enhance public health emergency preparedness (PHEP) and build community resilience. However, little research has focused on understanding this emerging field and its implications for PHEP. To address research gaps, this paper: (1) assesses the state of disaster citizen science by developing an inventory of disaster citizen science projects; (2) identifies different models of disaster citizen science; and (3) assesses their relevance for PHEP.

**Methods:**

We searched the English-language peer-reviewed and grey literature for disaster citizen science projects with no time period specified. Following searches, a team of three reviewers applied inclusion/exclusion criteria that defined eligible disasters and citizen science activities. Reviewers extracted the following elements from each project: project name and description; lead and partner entities; geographic setting; start and end dates; type of disaster; disaster phase; citizen science model; and technologies used.

**Results:**

A final set of 209 projects, covering the time period 1953–2017, were included in the inventory. Projects were classified across five citizen science models: distributed or volunteer sensing (*n* = 19; 9%); contributory (*n* = 98; 47%); distributed intelligence (*n* = 52; 25%); collaborative research (*n* = 32; 15%); and collegial research (*n* = 8; 4%). Overall, projects were conducted across all disaster phases and most frequently for earthquakes, floods, and hurricanes. Although activities occurred globally, 40% of projects were set in the U.S. Academic, government, technology, and advocacy organizations were the most prevalent lead entities. Although a range of technologies were used, 77% of projects (*n* = 161) required an internet-connected device. These characteristics varied across citizen science models revealing important implications for applications of disaster citizen science, enhancement of disaster response capabilities, and sustainability of activities over time.

**Conclusions:**

By increasing engagement in research, disaster citizen science may empower communities to take collective action, improve system response capabilities, and generate relevant data to mitigate adverse health impacts. The project inventory established a baseline for future research to capitalize on opportunities, address limitations, and help disaster citizen science achieve its potential.

## Background

As disasters become increasingly costly due to factors such as population growth, the important role of communities and individuals in public health emergency preparedness (PHEP) has gained societal prominence [1–4]. In 2011, the Centers for Disease Control and Prevention (CDC) included community preparedness, which entailed community engagement and partnership development, as one of the core public health preparedness capabilities for state and local health departments [1]. In the same year, the Federal Emergency and Management Agency (FEMA) issued their framework on a “whole community approach” to emergency management, where multiple stakeholders (e.g., residents, community leaders, government) work together to strengthen capacities and build community resilience [2]. In the last decade, there has been growing encouragement of bystander response and the involvement of individuals in activities historically left to first responders and government (e.g. groups such as the Cajan Navy) [5, 6]. Given that disasters will always be a reality,, developing strategies for promoting community involvement in disaster preparedness should continue to be a public health and national security priority.

Against this backdrop, a *citizen science* movement for disaster preparedness has also emerged. Citizen science is the use of scientific principles and methods by “non-professional” scientists or public volunteers to explore or understand the world around them [7]. In addition to preparedness, citizen science has proliferated across scientific disciplines due to factors such as the growing accessibility of measurement and monitoring tools, ubiquity and increased computing power of mobile devices, and governmental and academic encouragement [8]. The potential benefits of disaster citizen science for improving PHEP are numerous. Through engagement in scientific activities, citizen scientists may help stretch resources and enhance governmental responses through the timely collection of local-level data. Disaster citizen science may be empowering, helping communities build social networks, develop skills, and generate data to mitigate adverse disaster impacts. Communities may therefore gain knowledge and capacity to take actions, better respond and adhere to preparedness recommendations, and increase their resilience, or ability to bounce back from disaster events.

Citizen science has a long history in fields like ecology, with discussions surrounding its uses as part of the scientific discourse [9]. In contrast, while there is an extensive literature on spontaneous and organized volunteerism in disasters, little research to date has focused on understanding the field of disaster citizen science and the use of volunteers specifically for disaster citizen science activities. As a result, there are few materials or guiding principles from which to draw lessons to support the implementation of citizen science for PHEP. Additionally, lessons learned from citizen science in other fields may not generalize to disaster settings because they are often dangerous. The chaotic environments accompanying disasters may pose risks to citizen scientists, and the integration of these activities with official response and recovery functions may not be straightforward. Therfore, research is needed to assess the overall state of disaster citizen science and draw out implications for the use and conduct of citizen science in PHEP applications. To address research gaps, we aimed to: (1) assess the state of disaster citizen science by developing an inventory of disaster citizen science projects; (2) identify and describe different models of disaster citizen science; and (3) assess implications for different disaster phases. The construction of this first-ever comprehensive inventory will facilitate the identification of lessons learned that may increase the utility and value of disaster citizen science and improve system response capabilities, citizen scientist activities, and the resilience of affected communities.

## Methods

To construct the disaster citizen science inventory, we: (1) developed a search strategy; (2) applied inclusion/exclusion criteria; and (3) performed data extractions and analyses.

### Search strategy

#### Data sources

We reviewed the peer-reviewed and grey (e.g., white papers, technical reports) literature for disaster citizen science projects or activities (hereafter referred to as projects). As citizen science crosses a range of disciplines (e.g., ecology, sociology, biomedical, public health, engineering), we searched different databases represented multiple disciplines. For peer-reviewed literature, we searched PubMed, EBSCOhost research databases, Web of Science, Scopus, ArticleFirst, and OCLC Online Computer Library Center Electronic Collections Online. For grey literature, we searched LexisNexis, citizen science project databases and websites (see Additional file 1: Table S1), and Google (first ten pages of hits per search term). We also solicited feedback from experts and stakeholders by emailing a request for disaster-related citizen science projects on the Citizen Science Association listserv.

#### Search terms and restrictions

For the peer-reviewed literature, our search terms used “citizen science” terms AND “disaster” terms. For citizen science, we used multiple terms to capture the concept of non-professionals or volunteers engaging in research (e.g., citizen scien*, community scien*) [7]. For disasters, we included hazards identified as causing a public health emergency or a FEMA disaster declaration in the U.S. prior to 2018 [10, 11]. We also included climate change given its prominence as a national health security issue of concern [12]. (Additional file 2: Table S2) presents the complete list of search terms for the peer-reviewed literature.

Due to the large size of the grey literature, use of all citizen science terms yielded tens of thousands of returns. Therefore, for this literature, we paired each disaster term with the phrase “citizen science,” “community science,” or “crowdsourcing.”

For all databases, we restricted searches to titles, abstracts, or keywords, and only searched English language sources. We did not restrict searches by date to be as comprehensive as possible and ensure inclusion of projects that may lend historical perspective, and our review reflects the literature published prior to 12/31/2017.

This process yielded more than 2800 articles, websites, and emails that we scanned for disaster citizen science projects. Identified projects (*n* = 353) were then screened for inventory eligibility.

### Inclusion/exclusion screening

A two-step screening process was applied to the 353 projects. First, we applied a taxonomy developed by Wilderman to determine citizen science relevance for the inventory [13]. In Wilderman’s taxonomy, citizen science models are characterized by volunteer engagement in the following research activities: (1) problem definition; (2) study design; (3) sample/data collection; (4) data analysis; and (5) data interpretation. We included a project if volunteers were involved in one or more of these activities, with one caveat. If volunteers “collected” data on themselves (e.g., disease symptoms) (#3), then we also required involvement in at least one other activity for a project to be considered relevant. We chose this interpretation to guard against the inclusion of projects that involve citizens mainly as research subjects or inputs.

Second, we excluded projects that focused on routine monitoring activities (e.g., air or water quality) unless an activity was tied to a specific disaster event. Routine monitoring is vital for PHEP but raises conceptual issues about the demarcation between PHEP and routine public health functions. Therefore, we excluded monitoring projects to draw clear boundaries around disaster citizen science.

Three project team members carried out the screening process through a phased approach. Two team members applied inclusion/exclusion criteria to a set of assigned projects while the third person (the lead author) reviewed all projects and engaged in discussions to help resolve disagreements. Four rounds of screening and review occurred. By the last round of screening, reviewers had achieved a satisfactory level of agreement for including or excluding projects prior to team discussions (87% observed proportionate agreement, kappa 0.7). Following screenings, a final set of 209 projects were eligible for the inventory.

### Data extraction and analysis

We extracted the following data elements on each project (see Table 1 for more detailed descriptions): (1) project name and description; (2) lead project entities and entity type; (3) partners and other involved entities; (4) geographic setting; (5) project start and end years; (6) disaster type of focus; (7) disaster phase; (8) citizen science types; (9) citizen scientist participant roles; and (10) types of technologies used. Citizen science types and citizen scientist participant roles were considered two classification schemes for describing citizen science activities. In addition, we developed categories of project objectives through assessment across the entire dataset. We did not assign objectives to individual projects because of the difficulties of obtaining reliable information through review of project materials alone. For example, projects carried out to collect data for public health surveillance purposes may also have integrated educational or social networking components. Unless explicitly stated in existing materials however, it was not clear what project leaders would define as the intended objectives.

**Table 1:** Description of extraction elements for the project inventory

Element	Description and categorizations
Project name and description	Formal name of project and description of objectives
Lead project entities and entity type	Lead organization(s) or individuals for the project: academic/research; government; advocacy or issues-based; community-based services; volunteer or relief services; professional association; health services; technology sector; collaborative entity; individuals/loose affiliations
Partners and other involved entities	Listing of partners or entities cited by the project (if available)
Geographic setting	U.S., international, or global focus. If U.S., region specified (northeast, southeast, midwest, west, southwest, national (all)). If international, continent specified (North America, South America, Europe, Asia, Africa, Australia, Antarctica)
Project start and end years	Official year of project launch and end year (or ongoing)
Disaster type	Disaster(s): accidental explosion/fire; harmful algal bloom/cyanobacteria; drought; earthquake; flood; chemical contamination; hurricane/typhoon/cyclone; disease outbreak; technological failure; mud/landslide; nuclear radiation; severe storm/weather; terrorism; tornado; tsunami; volcanic activity; wildfire; all hazards; other
Disaster phase	Preparedness (pre-disaster, prevention and preparation activities dominate); response (during or in the immediate aftermath of the disaster, crisis activities dominate); recovery (post-disaster, rebuilding activities dominate); all phases
Citizen science types	Citizen science type based on the level of volunteer involvement:^a^ ○ *Contributory.* Volunteers involved mainly in data collection or reporting for projects led by professional scientists. ○ *Collaborative or co-created.* Volunteer and professional scientists working together on many aspects of the research for projects led by either group. ○ *Collegial.* Volunteers leading all aspects of the research with little participation by professional scientists.
Citizen scientist participant roles	Roles: (1) data collectors or reporters; (2) data interpreters and/or analyzers; and/or (3) problem definition and/or study design
Type of technologies used	Technologies used by volunteers: internet-connected device; communication device (e.g., phone, text, fax, radio); online forms/survey tools; crowdsourcing reporting applications (allows users to report or submit information); crowdsourcing analytical applications (allows users to engage in analytical tasks); mapping platforms/technologies; camera/video; sampling equipment/monitors/sensors; do-it-yourself sampling equipment; analytical software or tools; none; other; unknown

^a^Framework for citizen science type adopted from: Shirk et al. [14]

Four project team members performed extractions. The team used a variety of materials to obtain project information including original source documents and supplemental Google searches. Before independent extractions, the team applied a coding guide to a common set of projects (*n* = 15). The team met to discuss and resolve any differences and modify the coding guide as necessary. After trainings, three team members independently performed extractions for assigned projects while the fourth (lead author) reviewed all extractions.

## Results

**Fig. 1:**
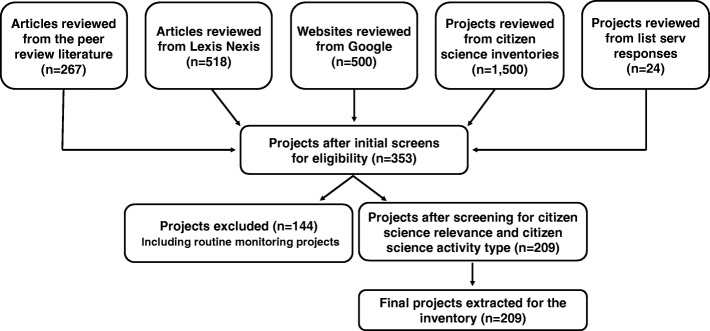
Flow diagram for project inventory development. Following eligibility review of over 2800 articles, websites, and potential projects, 353 potential projects were identified. Screening for citizen science relevance and removal of monitoring projects resulted in 209 projects included in the final inventory

Fig. 1 displays the flow diagram and search results. Most of the final 209 projects were identified through Google (*n* = 153), followed by the peer-reviewed literature (*n* = 64), citizen science inventories and websites (*n* = 26), list serv responses (*n* = 22), and LexisNexis (*n* = 10). Counts include projects overlapping multiple sources. Table 2 provides a summary of the data extracted for each project. (Additional file 3: Table S3) displays the complete project inventory along with extracted data for each data element.

**Table 2:** Disaster citizen science projects grouped by citizen science model

Project name	Description of citizen science or volunteer activities	Location	Dates	Disaster
Distributed sensing projects (*n* = 19)				
*Seismometers in school projects*				
1. IRIS networks	Create an international seismometer network in K-16 classrooms	Global	2001-	EQ
2. Nera Project	Create a European network of school seismometers	Global	2011-	EQ
3. Jamaican Educational Seismic Network	Understand Jamaica’s seismic risk through school-based seismic network	N Am	2016-	EQ
4. EduSeis	Create a school-based earthquake monitoring system in four European countries	Europe	1996-	EQ
5. O3EProject	Create a seismometer network in European schools	Europe	2007–09	EQ
6. Seismo at School	Create a seismometer network in Swiss schools	Europe	2007-	EQ
7. Seismology in Schools (Seismeolaíocht sa Scoil)	Create a seismic network of primary and secondary educational sectors	Europe	2007-	EQ
8. SISMOS à l’Ecole	Place seismometers in schools to record regional or global seismic activity	Europe	2006-	EQ
9. UK School Seismology Project	School network detects and shares seismometer measurements	Europe	2007-	EQ
10. Seismometers in Schools	Create a seismometer network in Australian schools	Australia	2012-	EQ
11. Seismometers in Schools	Create a seismometer network in New Zealand schools	Australia	2013-	EQ
12. MiQuakes	Place seismometers in Michigan schools	US	2011-	EQ
13. Oregon Shakes	Place seismometers in Oregon schools	US	Current	EQ
14. Princeton Earth Physics Project	Pioneer the creation of seismic networks in U.S. schools	US	1994	EQ
*Other projects*				
15. MyShake	Create seismic network using smartphone sensors to report earthquake shaking	Global	2016-	EQ
16. Quake-Catcher Network	Sense seismic motion through a computer-connected sensor network	Global	2008-	EQ
17. Community Seismic Network	Monitor earthquakes through a computer-connected network of sensors	US	2011-	EQ
18. NetQuakes	Install seismographs in areas with broadband internet connection	US	2009-	EQ
19. weather@home	Run regional climate modeling experiments on network of volunteer computers	Global	2010-	CL
Contributory projects (*n* = 98)				
*Seismic surveys*				
1. LastQuake (International)	Report earthquake observations through online or mobile applications	Global	2014-	EQ
2. Have You Felt an Earthquake, UK		Europe	2003	EQ
3. Other European countries (*n* = 26)		Europe	Current	EQ
4. Felt It (New Zealand)		Australia	2001-	EQ
5. Did You Feel It? (US)		US	1997-	EQ
*Other projects*				
6. Impacts of 2010 Haiti earthquake	Community workers conduct health surveys in the Haitian diaspora	US	2010	EQ
7. Cazadores de Crecidas, Argentina	Estimate river discharges through videos and photos to help flood modeling	S Am	2014-	FL
8. CITHYD (Citizen Hydrology)	Collect water level data in Italian waterbodies	Europe	2016-	FL
9. FloodCrowd	Report floods and impacts	Europe	2015-	FL
10. The FloodScale Project	Provide or share home movies of flooding to use in modeling flash floods	Europe	2012–15	FL
11. The RiskScape Project	Provide photo reports of floods to develop flood hazard models	Australia	2014	FL
12. Community flood monitoring	Install rain gauges in volunteer homes	Asia	2009	FL
13. Flood hazard mapping, India	Use participatory mapping approaches to assess flood vulnerability	Asia	− 2014	FL
14. Flood Patrol (UP-NOAH)	Report floods and impacts to inform preparedness efforts, Philippines	Asia	2012-	FL
15. Jakarta floods (PetaJakarta)	Report flood events using social media	Asia	2014–15	FL
16. Flood, water monitoring, Kenya	Measure water levels in Sondu River basin, Kenya	Africa	2014-	FL
17. FLOCAST	Enable citizen flood reports to improve flash-flood predictions	US	2013-	FL
18. Citizen science for the El Nino	Report coastal flood impacts due to the 2015–2016 El Nino	US	2015–16	FL
19. Boulder flood	Use of crowdsourcing map to report flood/damage observations	US	2013	FL
20. Crowdmap	Post observations about geological exposures or hazards to online community	Europe	2011-	FL,LS
21. Crowdwater	Provide flood, drought reports to improve forecasts of hydrological events	Europe	2016-	FL,DR
22. Drought Information Supported by Citizen Scientists (DISCS)	Provide hydrologic and agricultural information to the scientific community	US	2017-	DR
23. DroughtWatch	Support local drought vulnerability assessments through reporting data	US	2009–14	DR
24. King Tides Project International	Report King Tides observations to understand flood risk in coastal areas	Global	2009-	FL,CL
25. MyCoast King Tides (*n* = 9)	Document King Tides to track sea level rise and impacts (in nine US states)	US	2014-	FL,CL
26. Urban Tides Initiative	Report tide observations to understand effects of sea level rise	US	2015-	FL,CL,SW
27. Phones and Drones	Provide photos, videos of coastal damage due to the 2015–16 El Nino in CA	US	2016	FL,CL,SW,HR
28. MyCoast StormReporter (*n* = 6)	Report coastal storm damages (in six US states)	US	2014-	FL,SW,HR
29. mPING	Collect weather information to improve weather predictions and forecasting	Global	2012-	FL,SW,HR, TD,LS
30. SKYWARN	Trained weather spotters report data to improve emergency warning services	US	1960-	FL,SW,HR,TD
31. Community Collaborative Rain, Hail, and Snow Network	Measure precipitation for drought and flood modeling and monitoring	US	1998-	FL,SW,DR
32. Community DustWatch	Monitor wind erosion and dust events in Australia	Australia	2002-	SW
33. Send Us your Dirt from Sandy	Send researchers dirt samples post-Superstorm Sandy for chemical analysis	US	2012–15	HR
34. SkyTruth Spill Tracker	Report pollution incidents from hurricanes occurring during the fall of 2017	US	2017-	HR
35. Waterisotopes.org	Collect precipitation during Superstorm Sandy and send to researchers	US	2012–13	HR
36. El Reno tornado survey	Contribute observations (photos, videos, visual reports) of the El Reno tornado	US	2013–15	TD
37. Report a Landslide	Provide reports of landslide observations in Great Britain	Europe	2008-	LS
38. Did You See It?	Contribute to a US database of landslide observations	US	2012–16	LS
39. Bloomin’ Algae!	Report HABs in the United Kingdom	Europe	2017-	HAB/CB
40. Algae Alert Network	Monitor for HABs in the St. Croix River, WI	US	2012-	HAB/CB
41. bloomWatch	Report cyanobacteria blooms	US	2010-	HAB/CB
42. Tracking algal blooms	Engage pilots to photo algal blooms in Lake Erie	US	2016-	HAB/CB
43. CyanoTRACKER	Facilitate public reports of cyanobacteria blooms in Georgia waterbodies	US	2015-	HAB/CB
44. HAB Watch	Create a HAB monitoring network in Southern California	US	2011-	HAB/CB
45. Measure the Muck	Measure waterbody contaminants after flooding that contribute to HABs	US	2017	HAB/CB
46. Smith River algae reports	Visitors photograph and report algae growth in the Smith River, MT	US	2017-	HAB/CB
47. Owasco Lake HAB monitoring	Monitor and sample for HABs in Owasco Lake, NY	US	2015-	HAB/CB
48. HAB monitoring (Multiple)	Integrate HAB monitoring into regular water quality monitoring activities	US	Current	HAB/CB
49. Forest fuels measurement	Report data on forest fuels observations for wildfire risk prediction	N Am	2012-	WF
50. Live fuel moisture monitoring	Measure moisture content in living plant tissue to predict wildfire risk	US	2013-	WF
51. Is Ash Falling?	Collect ashfall samples during volcanic eruptions	Global	2013-	VL
52. myVolcano	Collect ash samples during volcanic eruptions and report observations	Europe	2010-	VL
53. Global Mosquito Alert	Enact global surveillance and control of mosquito species	Global	2017-	DO
54. MosquitoWEB	Observe and send mosquitoes to researchers in Portugal	Europe	2014-	DO
55. Muggenradar (Mosquito Radar)	Observe and send mosquitoes to researchers in the Netherlands	Europe	2014-	DO
56. Animal mortality monitoring	Monitor and report animal deaths to prevent Ebola outbreaks	Africa	2001–03	DO
57. Oil Reporter	Report observations of oil spill hazards	US	2010–11	CH
58. Oil Spill Tracker	Report and track impacts of the Deepwater Horizon oil spill	US	2010–17	CH
59. Integrated Fukushima Ocean Radionuclide Monitoring Network	Monitor Canada’s oceans for radionuclides through seawater sampling	N Am	2014-	NR
60. Our Radioactive Ocean	Collect seawater samples to monitor radiation levels	US	2013-	NR
Distributed intelligence (*n* = 52)				
1. Digital humanitarian projects (*n* = 34)	Support disaster response efforts in real-time through analyzing large amounts of different types of data. Includes 34 deployments.	N Am, S Am, Europe, Asia, Australia, US, Africa, Oceania	2010–17	EQ,FL,HR, DO,VL,TR, DR
2. Fukushima Futaba 2011 Archive of Japan Disasters	Preserve memories of affected communities in Futaba, Japan and foster research	Asia	2013-	EQ,NR,TS
3. SHETRAN and River Watch group catchment monitoring	Implement community flood observation program in northeast England to support development of a catchment model	Europe	2013–16	FL
4. Storm Photo	Document and determine severity of flooding in California	US	2015-	FL
5. WeSenseIt	Create citizen flood observatories through use of sensor devices	Europe	2012–16	FL,CL,DR
6. Operation Weather Rescue	Transcribe old weather observations for climate modeling	Europe	2017-	SW
7. iCoast – Did the Coast Change?	Identify coastal changes following extreme storms	US	2014-	SW,HR
8. CycloneCenter	Estimate intensity of cyclones through satellite images	Global	2012-	HR
9. Agricultural recovery post-Hurricane Mitch	Enlist Nicaraguan farmers in assessing farming methods that could enhance disaster recovery	S Am	1999	HR
10. Rural Alaska Monitoring Program	Community monitoring for climate-mediated health threats	US	2014-	CL,HAB/CB
11. cyanoMonitoring	Monitor cyanobacteria populations over time	US	2010-	HAB/CB
12. cyanoScope	Understand where and when cyanobacteria species occur	US	2010-	HAB/CB
13. SoundToxins	Explore Puget Sound conditions that affect algal bloom events	US	2006-	HAB/CB
14. National Phytoplankton Monitoring Network	Monitor marine phytoplankton and algal blooms across the US	US	2001-	HAB/CB
15. Community volcano monitoring	Create network for volcano monitoring in Ecuador	S Am	2000	VL
16. Mosquito Habitat Mapper	Track mosquito larvae, eliminate breeding sites, and share data	Global	2017-	DO
17. Mosquito Alert	Track mosquitos, breeding sites, and validate shared photos	Europe	2014-	DO
18. Zanzamapp	Trap and report on mosquitoes in Italy	Europe	2016-	DO
19. Invasive Mosquito Project	Track invasive mosquito species across the US	US	2015-	DO
Collaborative research (*n* = 32)				
1. Maori response to Christchurch earthquakes	Understand how cultural attributes inform preparedness strategies	Australia	2010–15	EQ
2. Environmental Competency Groups	Demonstrate a method for collaborative investigation	Europe	2007-	FL
3. Flood Network	Create flood detection network in the United Kingdom	Europe	2014-	FL
4. Participatory water monitoring in Tanzania	Villagers collect and analyze data to address flood concerns	Africa	2001–11	FL
5. Environmental exposure survey, Atlanta	Document asthma and exposures in two flood-prone communities	US	2014	FL
6. Beacon of Hope M.O.D.E.L., Hurricane Katrina	Map recovery needs using community-led recovery framework	US	2006-	HR
7. Community mapping post-Katrina	Pastors address uneven redevelopment patterns post-Katrina	US	2007	HR
8. Health care needs in New Orleans post-Katrina	Engage community to understand healthcare needs post-disaster	US	2006	HR
9. Participatory action research post-Katrina	Use participatory photo approach to assess health experiences	US	2006–09	HR
10. Videovoice for recovery post-Katrina	Use participatory video approach to address issues of concern	US	2007–08	HR
11. Participatory research after Hurricane Floyd	Develop a survey to document displaced survivor experiences	US	2000–01	HR
12. PhotoVoice for disaster reduction strategies	Use participatory photo approach for vulnerability assessments	US	−2013	TS
13. Lake Winnipeg citizen science initiative	Monitoring algal bloom formation in Lake Winnipeg, Canada	N Am	2016-	HAB/CB
14. Lake Champlain volunteer monitoring	Document algal blooms in Lake Champlain	US	2004-	HAB/CB
15. Appalachian Water Watch	Report emergency water pollution events	US	2013-	HAB/CB, CH
16. Participatory action research in Australia	Investigate pandemic influenza risk in Indigenous communities	Australia	2007	DO
17. Mosquito Alert (Hong Kong)	Track mosquitos, breeding sites, and validate photos	Asia	2017-	DO
18. Understanding fishing communities	Address oil spill risks in Vietnamese-American fishing communities	US	2017-	CH
19. Consortium for oil spill exposure pathways	Address oil spill risks in Vietnamese-American fishing communities	US	2015-	CH
20. Monitoring oil contamination in Louisiana	Develop a citizen science oil spill monitoring program	US	2017-	CH
21. Oil Spill Crisis Map	Report and map impacts of Deepwater Horizon oil spill	US	2010-	CH
22. Flint water crisis	Test tap water for lead contamination in Flint, Michigan	US	2015–17	CH
23. Love Canal contamination	Perform health surveys to assess chemical contamination	US	1978–80	CH
24. The Buffalo Lupus project	Assess links between waste site exposure and autoimmune disease	US	2001–06	CH
25. Graniteville recovery & chlorine epidemiology	Address community recovery of Graniteville, SC post-chlorine spill	US	2005–15	CH
26. Tonawanda Coke Corporation pollution	Address exposure and health impacts resulting from pollution	US	2005–09	CH
27. Safecast	Map global radiation and build worldwide sensor network	Global	2011-	NR
28. Citizen Radioactivity Measuring Stations	Take radiation measurements and make judgments on risks	Asia	2011-	NR
29. Towa Organic Village, Japan and Fukushima	Villagers monitor radiation and perform collaborative research	Asia	2011-	NR
30. Nuclear Risk Management for Native Communities	Address nuclear contamination impacts in tribal communities	US	1994–04	NR
31. St. Louis baby tooth survey	Examine radioactive material absorbed into teeth of children	US	1958–70	NR
32. Hazelwood Mine fire recovery effort	Develop citizen science environmental monitoring program	Australia	2014-	EF
Collegial research (*n* = 8)				
1. Groninger Soil Movement	Monitor earthquakes and damage due to gas extraction	Europe	2009-	EQ
2. Queensland Floods	Use social media to provide data and reconstruct flood extents	Australia	2010	FL
3. Historic Extreme Weather Event Reporting	Research historical documents on extreme weather events	N Am	2016-	SW,HR,TD
4. Community water testing in Puerto Rico	Perform water testing in Puerto Rico post-Hurricane Maria	US	2017-	HR
5. VGI and Santa Barbara wildfires	Map and share social media data during 2007–09 wildfires	US	2008–09	WF
6. Gulf Oil Mapping Project	Map impacts after Deepwater Horizon oil spill	US	2010	CH
7. iWitness Pollution Map	Report and map chemical accident reports and impacts	US	2010-	CH
8. Young Crowd	Assess disaster preparedness of youth environments	Europe	2016–17	AH

*Abbreviations: S Am* South America, *N Am* North America, *EQ* earthquake, *CL* climate change, *FL* flooding, *SW* severe weather, *HR* hurricane, *HAB/CB* harmful algal blooms/cyanobacteria, *DR* drought, *TD* tornado, *LS* landslide, *DO* disease outbreak, *WF* wildfire, *VL* volcanic activity, *CH* chemical, *NR* nuclear radiation, *TR* terrorism, *TS* tsunami, *EF* explosion/fire, *AH* all hazards

References: See (Additional file 3: Table S3) for full project inventory and source references

### Disaster citizen science project objectives

Overall, the disaster citizen science projects reviewed in this study were designed to achieve many different objectives, including: assessment of risks or community vulnerabilities; surveillance, early-warning, and monitoring; database or repository building; historical research or baseline establishment; intervention development and testing; epidemiological investigations; and population needs assessments. In addition, beyond scientific objectives, projects could also be designed to achieve broader societal impacts that may yield benefits for enhancing community resilience such as performing outreach to isolated groups, providing education and raising awareness about hazards and impacts, or building networks through collaborative problem-solving [15].

### Disaster citizen science models

The two classification schemes describing citizen science activities together comprised a framework incorporating elements of typologies developed by Shirk et al. [14] and Haklay [16]. Using the new framework, projects were categorized into one of five citizen science models:

- **Distributed or volunteer sensing (*n*** **= 19; 9%).** Citizen scientists volunteer resources or space to facilitate data collection or analyses led by professional scientists.
- **Contributory (*n*** **= 98; 47%).** Citizen scientists collect data to assist research led by professional scientists.
- **Distributed intelligence (*n*** **= 52; 25%).** Citizen scientists perform data analyses or interpretation.
- **Collaborative research (*n*** **= 32; 15%).** Citizen and professional scientists collaborate in areas beyond data collection or analysis (e.g., problem definition, study design).
- **Collegial research (*n*** **= 8; 4%).** Citizen scientists lead research with little collaboration with professional scientists.

The next sections describe how these models of citizen science vary across project characteristics (see Table 3 for descriptive statistics).

**Table 3:** Frequencies of dataset characteristics by citizen science model

	Overall		Distributed sensing		Contributory		Distributed intelligence		Collaborative research		Collegial research	
	209		19	(9%)	98	(47%)	52	(25%)	32	(15%)	8	(4%)
Disaster												
Earthquake	61	(29%)	18	(95%)	31	(23%)	10	(17%)	1	(3%)	1	(10%)
Flood	52	(25%)			36	(26%)	11	(19%)	4	(12%)	1	(10%)
Hurricane, typhoon, cyclone	36	(17%)			12	(9%)	16	(28%)	6	(18%)	2	(20%)
Harmful algal blooms/cyanobacteria	18	(9%)			10	(7%)	5	(9%)	3	(9%)		
Severe/extreme weather	15	(7%)			12	(9%)	2	(3%)			1	(10%)
Climate change or sea level rise	15	(7%)	1	(5%)	12	(9%)	2	(3%)				
Chemical contamination events	14	(7%)			2	(1%)			10	(30%)	2	(20%)
Disease outbreak	11	(5%)			4	(3%)	5	(9%)	2	(6%)		
Nuclear radiation	8	(4%)			2	(1%)	1	(2%)	5	(15%)		
Drought	6	(3%)			4	(3%)	2	(3%)				
Mud/landslides	4	(2%)			4	(3%)						
Tornado	4	(2%)			3	(2%)					1	(10%)
Volcanic activity	4	(2%)			2	(1%)	2	(3%)				
Wildfire	3	(1%)			2	(1%)					1	(10%)
Tsunami	2	(1%)					1	(2%)	1	(3%)		
Lead entity												
Academic/research	94	(45%)	18	(95%)	49	(39%)	10	(18%)	15	(33%)	2	(18%)
Government	55	(26%)	1	(5%)	46	(37%)	6	(11%)	3	(7%)		
Technology	51	(24%)			16	(13%)	34	(62%)	1	(2%)		
Advocacy	23	(11%)			6	(5%)	1	(2%)	12	(26%)	4	(36%)
Collaboration	13	(6%)			5	(4%)	2	(4%)	6	(13%)		
Community-based services	6	(3%)			1	(1%)			4	(9%)	1	(9%)
Individuals/loose affiliation	5	(2%)			1	(1%)			2	(4%)	2	(18%)
Volunteer services	3	(1%)			1	(1%)	1	(2%)			1	(9%)
Education	3	(1%)			1	(1%)			1	(2%)	1	(9%)
Disaster phase												
Preparedness	135	(65%)	17	(50%)	81	(58%)	17	(27%)	16	(37%)	4	(33%)
Response	52	(25%)	2	(6%)	10	(7%)	34	(54%)	3	(7%)	3	(25%)
Recovery	105	(50%)	15	(44%)	49	(35%)	12	(19%)	24	(56%)	5	(42%)
Location												
Global	13	(6%)	5	(26%)	5	(5%)	2	(4%)	1	(3%)		
United States	84	(40%)	5	(26%)	44	(45%)	10	(19%)	21	(66%)	4	(50%)
Northeast	20	(24%)			13	(28%)	2	(15%)	5	(22%)		
Southeast	27	(32%)			9	(19%)	2	(15%)	13	(57%)	3	(75%)
Midwest	6	(7%)	2	(40%)	2	(4%)			2	(9%)		
Southwest	8	(10%)			5	(11%)	2	(15%)	1	(4%)		
West	18	(21%)	2	(40%)	10	(21%)	3	(23%)	2	(9%)	1	(25%)
National	13	(15%)	1	(20%)	8	(17%)	4	(31%)				
International	112	(54%)	9	(47%)	49	(50%)	40	(77%)	10	(31%)	4	(50%)
North America	11	(10%)	1	(11%)	2	(4%)	6	(15%)	1	(9%)	1	(25%)
South America	6	(5%)			1	(2%)	5	(13%)				
Europe	54	(48%)	6	(67%)	37	(76%)	7	(18%)	2	(18%)	2	(50%)
Asia	23	(21%)			4	(8%)	15	(38%)	4	(36%)		
Africa	7	(6%)			2	(4%)	4	(10%)	1	(9%)		
Australia	10	(9%)	2	(22%)	3	(6%)	1	(3%)	3	(27%)	1	(25%)
Oceania	2	(2%)					2	(5%)				
Technology												
Internet-connected device	159	(76%)	17	(47%)	81	(36%)	46	(32%)	7	(15%)	8	(32%)
Camera/video	59	(28%)			40	(18%)	7	(5%)	7	(15%)	5	(20%)
Crowdsourcing reporting application	54	(26%)	2	(6%)	39	(17%)	6	(4%)	3	(6%)	5	(20%)
Sampling equipment/monitors/sensors	49	(23%)	17	(47%)	13	(6%)	6	(4%)	12	(25%)	1	(4%)
Online form/survey	44	(21%)			38	(17%)	1	(1%)	3	(6%)	2	(8%)
Crowdsourcing analytical application	43	(21%)			1	(0.4%)	40	(28%)	1	(2%)	1	(4%)
Mapping platforms/technologies	38	(18%)			2	(1%)	32	(23%)	3	(6%)	1	(4%)
Communication device	11	(5%)			6	(3%)	1	(1%)	3	(6%)	1	(4%)
Do-it-yourself sampling equipment	6	(3%)			4	(2%)	1	(1%)			1	(4%)
Lab equipment	2	(1%)					2	(1%)				
None	8	(4%)			1	(0.4%)			7	(15%)		

#### Citizen science models by disaster type

Overall, citizen science projects were carried out most frequently for earthquakes (*n* = 61; 29%), floods (*n* = 52; 25%), and hurricanes (*n* = 36; 17%). Disaster types varied across citizen science model. Earthquakes comprised the bulk of distributed sensing projects (*n* = 18; 95%). Earthquakes (*n* = 31; 32%) and floods (*n* = 36; 37%) were the main disasters for contributory projects. The majority of distributed intelligence projects focused on earthquakes (*n* = 10; 19%), floods (*n* = 11; 21%), or hurricanes (*n* = 16; 31%). Most collaborative research projects were focused on nuclear radiation (*n* = 5; 16%), hurricanes (*n* = 6; 19%), and chemical contamination events (*n* = 10; 31%). Finally, half of collegial research projects addressed either hurricane (*n* = 2; 25%) or chemical contamination events (*n* = 2; 25%).

#### Citizen science models by lead and collaborating entities

Most projects were led by academic/research groups (*n* = 94; 45%) followed by government (*n* = 55; 26%), technology groups (organizations focused on development or deployment of technological resources, such as equipment or online platforms) (*n* = 51; 24%), and advocacy organizations (*n* = 23; 11%). We also collected the names of listed partners for each project, but it was often difficult to determine the role of every partner or the extent of their involvement. The majority of projects (*n* = 160; 77%) listed at least one partner. When identified, partners provided different types of services or support including: funding, technical assistance, equipment, digital platforms, manpower, administrative support, or evaluation capabilities.

Across models, academic groups led a large proportion of distributed sensing (*n* = 18; 95%), contributory (*n* = 49; 50%), and collaborative research (*n* = 15; 47%) projects. Government was primarily involved as lead for contributory projects (*n* = 46; 47%). Technology groups led the greatest proportion of distributed intelligence projects (*n* = 34; 65%). Advocacy organizations showed a greater lead role in collaborative (*n* = 12; 38%) and collegial research (*n* = 4; 50%) projects compared to the other models. Finally, partnerships led 19% (*n* = 6) of collaborative research projects, with academic and community organizations or a coalition of community groups most often comprising the partnership.

#### Citizen science models by disaster phase

Projects covered all disaster phases includingpreparedness (*n* = 135; 65%), response (*n* = 52; 25%), and recovery (*n* = 105; 50%), and some covered more than one phase. Distributed sensing was more likely to be focused on preparedness (*n* = 17; 89%) and recovery (*n* = 15; 79%) versus response (*n* = 2; 11%). Contributory projects focused on preparedness (*n* = 81; 83%) and recovery (*n* = 49; 50%). In contrast, the distributed intelligence model was most often used for response (*n* = 34; 65%). The recovery phase comprised 75% (*n* = 24) of collaborative research projects compared to 50% (*n* = 16) for preparedness and 9% (*n* = 3) for response. Most collegial research projects focused on recovery (*n* = 5; 63%).

#### Citizen science models by geographic setting

Projects were implemented globally, with 40% (*n* = 84) of projects set in the U.S. and 54% (*n* = 112) implemented outside the U.S. Thirteen (6%) projects were global in nature with no specific focus on any one country or region of the world.

Distributed sensing projects showed greater international versus U.S. prevalence (*n* = 9; 47% and *n* = 5; 26%, respectively), while contributory projects were more evenly distributed across U.S. and international settings (*n* = 44; 45% and *n* = 49; 50%, respectively). For distributed intelligence, projects were more prevalent internationally (*n* = 40; 77%) than in the U.S. (*n* = 10; 19%). In contrast, collaborative research was more prevalent in U.S. (*n* = 21; 66%) versus international projects (*n* = 10; 31%). Collegial research projects were distributed evenly across U.S. and international settings. We also noted a few regional patterns. In the U.S., the contributory model comprised most projects across regions, with one exception. In the southeast, collaborative research was the most prevalent model type (*n* = 13; 48%). Internationally, the contributory model comprised the majority of projects in Europe (*n* = 37; 69%) and Australia (*n* = 3; 30%). However, for all other continents, distributed intelligence was most prevalent.

#### Disaster citizen science technologies

The majority of projects (*n* = 159; 76%) required an internet-connected device to perform research. Most frequently used technologies included: crowdsourcing applications (*n* = 94; 45%); cameras or video (*n* = 59; 28%); sampling, monitoring, or sensor equipment (*n* = 49; 23%); online survey tools (*n* = 44; 21%); and mapping platforms (*n* = 38; 18%).

Distributed sensing projects relied heavily on sensor equipment (*n* = 17; 89%). The majority of contributory projects used cameras or video (*n* = 40; 41%), crowdsourcing data reporting applications (*n* = 39; 40%), or online surveys (*n* = 38; 39%). Distributed intelligence projects used crowdsourcing data analysis applications (*n* = 40; 77%) and mapping technologies (*n* = 32; 62%). For collaborative research, sampling equipment (*n* = 12; 38%) and cameras or video (*n* = 7; 22%) were the most prevalent technologies. Finally, most collegial research projects used crowdsourcing reporting applications (*n* = 5; 63%) and cameras or video (*n* = 5; 63%).

#### Disaster citizen science trends

**Fig. 2:**
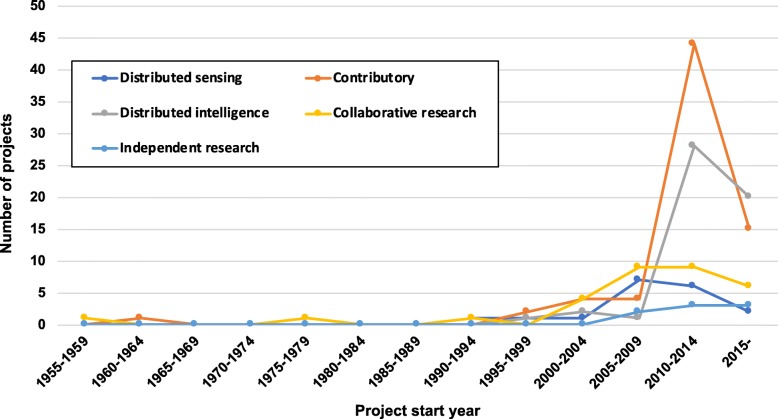
Number of disaster citizen science projects over time. Trends in incidence of projects grouped by five-year categories (starting from 1955 to ongoing projects as of 12/31/2017) are shown for each citizen science model (distributed sensing, contributory, distributed intelligence, collaborative research, and collegial research)

Figure 2 displays the incidence of disaster citizen science projects across years, and shows an upward trend beginning in the late 2000s for all models. Contributory and distributed intelligence models showed similar trends around the same time period. For the 164 projects with both start and end date information, 67 (41%) had ended while 97 (59%) were ongoing at the time of data capture. Across models, 79% (*n* = 15) of distributed sensing projects were ongoing, compared to 51% (*n* = 50) for contributory, 25% (*n* = 13) for distributed intelligence, 47% (*n* = 15) for collaborative research, and 50% (*n* = 4) for collegial research. Projects lasted from as little as a few weeks to as long as almost 58 years. For concluded projects, average duration was 1.3 years with a range of less than a year to 12 years.

## Discussion

Disaster citizen science is a rich field, comprised of diverse projects addressing many types of disasters and disaster phases. The field is growing worldwide, fueled by the use of digital technologies, and attracting multiple types of participants, including citizen volunteers, academics, government, and technology and advocacy sectors. Below we discuss four themes that arose from our assessment of the inventory. Patterns indicated potential differences across citizen science models in terms of: (1) addressing different disaster types; (2) enhancing activities across disaster phases; (3) use of technologies; and (4) exhibiting sustainability over time.

### Citizen science models and disaster type

Inventory analysis revealed patterns regarding the types of disasters addressed across different citizen science models. Distributed sensing, contributory, and distributed intelligence models were mainly focused on three disaster types – earthquakes, floods, and hurricanes. In these models, which are primarily led by professional scientists, citizen scientists were involved primarily in data collection or analysis, allowing for activities such as monitoring for an event, investigating disaster impacts, or providing information to aid response.

In contrast, collaborative and collegial research models, where citizen scientists have greater roles in leading, designing, and implementing activities, showed a larger focus on chemical contamination events – a type of technological disaster. Technological disasters are anthropogenic in origin and caused by the failure of manmade systems [17–19]. Research indicates that these events are characterized by a prolonged duration, uncertain effects; distrust in authorities; and identifiable parties to blame [20]. Chemical contamination disasters may be more likely to motivate community-led actions due to a perception of failure on the part of government or other institutions to protect the public from harm. As a result, beyond collecting data to aid in PHEP actions, collaborative and collegial research models may also provide a means for communities to channel frustrations, hold institutions accountable, engage in advocacy and problem-solving, and ensure involvement in decision-making processes. Professional scientists who engage with citizen scientists in collaborative and collegial research models may require extra training and skills beyond what is typically provided in graduate programs. Some may be reluctant to engage in these models due to concerns that citizen scientists will have a particular agenda, limited control over data collection and quality, and the extra time and resources required to navigate complex relationships with community members and organizations [9].

### Citizen science models and disaster phase

Certain models may be better suited to particular disaster phases. Distributed sensing and contributory projects focused primarily on preparedness, generally employing crowdsourced data to inform activities such as surveillance of human or environmental conditions. Given the low level of interaction and maintenance required by volunteers, distributed sensing may be a sustainable way to collect data or enhance analytical capacity.

Contributory models, along with distributed intelligence forms of citizen science, also seem well-suited to the response phase where there is need for real-time, local information about conditions. The distributed intelligence model in particular, has allowed a new form of disaster relief operations, termed “digital humanitarianism,” where volunteers away from a disaster site assist in digitally evaluating large amounts of information about the disaster (e.g., hotline requests, satellite imagery) [21]. As indicated by inventory projects, such approaches may be particularly beneficial for assisting resource-poor areas in disaster response where existing governmental or institutional structures may be inadequate to support a robust response on their own (e.g., earthquakes in Haiti, Pakistan; flooding in India, Sri Lanka; Ebola in West Africa).

Digital humanitarianism is filling a critical response need [21], but efforts are still needed to improve collection of timely, local-level data within disaster-affected areas. Federal agencies such as CDC and others have undertaken initiatives to make scientific research a part of disaster response, but there are challenges related to logistics, infrastructure, identification of research questions, and data quality [22–24]. However, inventory projects suggest that citizen science could help address some of these issues. For instance, after both the 2010 Deepwater Horizon oil spill and the 2011 Fukushima nuclear power plant meltdown, grassroots organizations led activities to create accessible tools and approaches such as apps that would allow individuals to measure and monitor disaster impacts in the environment [25, 26].

Finally, collaborative and collegial research projects tended to focus on the recovery phase. Collaborative research represents a somewhat more intensive project in terms of the need to develop and maintain strong partnerships, and could be highly valuable for inclusion of community needs for recovery planning and long-term recovery efforts. Given its nascency, collegial research is currently a more variable model in terms of objectives and structure. Collegial projects ranged from citizen scientists performing water quality testing to advocacy organizations spearheading crowdsourcing projects to track disaster impacts. Overall though, collegial research models afford communities the highest latitude in directing research to address community needs.

### Citizen science models and use of technologies

The majority of projects used some form of digital technology, particularly smartphones with dedicated data collection applications and sharing mechanisms, and we note that the growth of disaster citizen science tracks with technological milestones such as the launch of social media (~ 2004–2006) and release of mobile smartphones to the mainstream consumer market (~ 2007–2008). According to the Pew Research Center, in 2016, 77% of Americans owned a smartphone, 73% had home broadband service, and 69% of adults reported being social media users [27]. Globally, smartphone usage in developing countries increased from 21% in 2013 to 37% in 2015 [28]. Growing technology adoption may enhance accessibility and fuel opportunities for scientific engagement through data collection and sharing activities.

While citizen science models rely heavily on internet-connected devices, distributed sensing and distributed intelligence projects were reliant on a few specific types of technologies (crowdsourcing applications, sensors/monitors). In contrast, contributory, collaborative, and collegial research projects incorporated a greater range of technologies (e.g., cameras, video, crowdsourcing applications, sampling equipment, online forms or survey tools).

Although a promising trend overall, there are reasons to be cautious about overreliance on digital technologies during disasters. Events such as Superstorm Sandy and Hurricane Harvey did not result in destruction of the communications or electronic infrastructure necessary to utilize internet-enabled devices. However, as seen in Puerto Rico following Hurricane Maria, this might not always be the case. Citizen science efforts should take into account how best to use different forms of technology to ensure resilient systems and which models may be best able to facilitate actions when technology is limited.

### Citizen science models and project sustainability

Whether a project is sustained depends partly upon its intended goals. For example, 94% of distributed sensing projects were ongoing; this aligns with their focus on preparedness activities, which are often continuous in nature. In contrast, only 27% of distributed intelligence projects continued past a disaster event. However, the bulk of these projects were response-related and therefore time-limited in scope.

We note some cases however, where sustained efforts have grown organically and evolved over time. Community-led movements following the Deepwater Horizon oil spill and the Fukushima disaster led to the creation of Public Lab in the Gulf South [29], which now helps communities address their own scientific questions, and Safecast, which maintains the largest open dataset of background radiation measurements from across the globe [26]. Similarly, the 2010 Haiti earthquake catalyzed the digital humanitarianism movement, serving as the first case of a large-scale, collaborative effort between technology and humanitarian relief sectors [30]. These projects suggest a potential for sustainable citizen science models, where projects could shift objectives to address different disasters or disaster phases.

### Limitations

Although we carried out a comprehensive scan of the literature, our searches only included projects that were described or conducted in English and we were limited to describing activities based on publicly available data. In addition, it was difficult at times to draw definitive boundaries around certain disaster citizen science concepts. For example, we excluded routine monitoring activities that were not directed towards a specific disaster event. Other exclusions that could be considered disaster citizen science included environmental justice projects that addressed toxic pollution concerns and climate change projects that focused on ecological rather than human impacts (e.g., invasive species, coastal erosion). Finally, we note two definitional limitations. First, our inclusion criteria for a disaster omitted rare, emerging, or slow-moving events (e.g., food security, antimicrobial resistance). Second, our designations of lead and partner entities might not always be accurate as it was often difficult to discern these characteristics from literature sources alone.

## Conclusions

The public health impacts of disasters are significant: death, disease, injury, damage to homes and communities, and adverse mental and physical consequences. Given disasters will continue to strike, public health agencies are in need of tools to support PHEP efforts. Results from this first comprehensive inventory of disaster citizen science activity suggest that citizen science approaches are widely used and represent many areas of opportunity for PHEP. Disaster citizen science projects have the potential to expand PHEP capabilities such as facilitating greater data collection opportunities to support situational awareness, community risk and vulnerability assessments, and identification of recovery needs, if guidance on engaging in citizen science is made readily available to public health professionals.

The cataloguing of projects allows for a better understanding of the breadth of the field so those interested in initiating or participating in a disaster citizen science activity can find resources to tap into or leverage. Future research should explore the advantages and disadvantages of each citizen science model, barriers faced by the public health community in applying these models to different disaster contexts, promising implementation approaches, and strategies to support the proliferation of citizen science activities. In addition, more research is needed to understand the public health impacts of disaster citizen science projects, and whether and how, citizen science has demonstrably led to enhanced resilience. Our work represents a keystep in developing this understanding so that disaster citizen science achieves its potential to advance research, enhance community preparedness, and build community resilience for all.

## Supplementary information

**Additional file 1:** **Table S1.** Citizen science databases or websites used to identify disaster-related activities.

**Additional file 2:** **Table S2.** Search terms employed in peer-reviewed literature database searches.

**Additional file 3:** **Table S3.** Complete project inventory dataset.

## Data Availability

All data generated or analyzed during this study are included in this published article [and its additional information files].

## Abbreviations

AH: All hazards
CDC: Centers for Disease Control and Prevention
CH: Chemical
CL: Climate change,
DO: Disease outbreak
DR: Drought
EF: Explosion/fire
EQ: Earthquake
FEMA: Federal Emergency Management Agency
FL: Flooding
HAB/CB: Harmful algal blooms/cyanobacteria
HR: Hurricane
LS: Landslide
N Am: North America
NR: Nuclear radiation
S Am: South America
SW: Severe weather
TD: Tornado
TR: Terrorism
TS: Tsunami
VL: Volcanic activity
WF: Wildfire
