# Supplementary material for: Enhancing community preparedness: an inventory and analysis of disaster citizen science activities
Source: BMC Public Health. 2019 Oct 23;19:1356. doi: 10.1186/s12889-019-7689-x (PMC6813061; doi:10.1186/s12889-019-7689-x)
Supplement: Supplementary file 1 — Additional file 1: Table S1. Citizen science databases or websites used to identify disaster-related activities. [file 12889_2019_7689_MOESM1_ESM.docx]

**Table S-1.** Citizen science databases or websites used to identify disaster-related activities

| Database | Link |
| --- | --- |
| Citizen Cyberlab | http://www.citizencyberlab.org/ |
| Citizen Science Center | http://www.citizensciencecenter.com/ |
| CitSci.org | http://citsci.org |
| Center for Citizen Science | https://ccsorg.wordpress.com/about/ |
| Crowd4U | https://crowd4u.org/en/projects |
| Crowdcrafting | https://crowdcrafting.org/ |
| CrowdCurio | https://www.crowdcurio.com/ |
| Curious Minds | https://www.curiousminds.nz/ |
| Federal Crowdsourcing & Citizen Science Catalog | https://ccsinventory.wilsoncenter.org/  https://www.citizenscience.gov/ |
| iNaturalist | https://www.inaturalist.org/ |
| National Geographic | https://www.nationalgeographic.org/idea/citizen-science-projects/ |
| Scientific American | https://www.scientificamerican.com/citizen-science/ |
| SciStarter | http://scistarter.com |
| Wikipedia | https://en.wikipedia.org/wiki/List_of_citizen_science_projects  https://en.wikipedia.org/wiki/List_of_crowdsourcing_projects |
| World Community Grid | https://www.worldcommunitygrid.org/discover.action |
| Zooniverse | https://www.zooniverse.org/ |
