# Supplementary material for: Enhancing community preparedness: an inventory and analysis of disaster citizen science activities
Source: BMC Public Health. 2019 Oct 23;19:1356. doi: 10.1186/s12889-019-7689-x (PMC6813061; doi:10.1186/s12889-019-7689-x)
Supplement: Supplementary file 2 — Additional file 2: Table S2. Search terms employed in peer-reviewed literature database searches. [file 12889_2019_7689_MOESM2_ESM.docx]

**Table S-2.** Search terms employed in peer-reviewed literature database searches*

| Term type | Terms |
| --- | --- |
| Citizen science terms | citizen scien*, community scien*, public scien*, amateur scien*, civic scien*, participatory science, democratizing science, open science, informal science, activist science, popular science, street science, public participation in scientific research, community monitor*, participatory monitor*, volunteer monitor*, citizen observator*, crowdsourc*, popular epidemiology, participatory action research, community based participatory research, maker movement, participatory GIS, participatory geographic information system, VGI, volunteered geographic information |
| Disaster terms | disaster, preparedness, disaster recovery, disaster response, emergency preparedness, emergency response, community resilience, disaster resilience, humanitarian, crisis, catastrophe, famine, climate change, global warming, severe weather, extreme weather, severe storm, extreme event, hurricane, earthquake, seism, wildfire, flood, tornado, volcano, pandemic, epidemic, influenza, outbreak, ebola, landslide, avalanche, tsunami, monsoon, nuclear, radiation, radiological, drought, harmful algal blooms, chemical contamination, toxic contamination, water crisis, pollution crisis, oil spill, chemical spill, poison, explosion, accident, terrorism, bioterrorism, cyberterrorism, foodborne, waterborne, evacuation |

*Searches also included variations on terms (e.g., plurals, use of operators such as NEAR or WITHIN)
